# Developing the Next Generation of Physician Changemakers: “You Have to Love the People, and Love the Process”

**DOI:** 10.7759/cureus.84861

**Published:** 2025-05-26

**Authors:** Andrea L Austin, Anne Wildermuth, Joshua Hartzell, Jerusalem Merkebu

**Affiliations:** 1 Emergency Medicine, Uniformed Services University of the Health Sciences, Bethesda, USA; 2 Health Professions Education, Uniformed Services University of the Health Sciences, Bethesda, USA; 3 Medicine, Uniformed Services University of the Health Sciences, Bethesda, USA

**Keywords:** change, culture, emotional regulation, emotions, leadership

## Abstract

Introduction: There is a growing consensus among patients, physicians, and policymakers that healthcare must undergo a massive transformation to promote healthcare worker well-being. The Institute for Health developed the Triple Aim framework for high-performing health organizations, which focused on reducing costs, improving individual patient experience, and population health. The move from the Triple to Quadruple Aim added a focus on the well-being of healthcare professionals, acknowledging growing literature and a movement that healthcare professionals' well-being is crucial to the quality of healthcare patients receive.

Objectives: This study sought to investigate the individual transformation and the organizational contributors that promote effective change. More specifically, how do attending physicians from various specialties construct their understanding and make sense of the individual and organizational factors that contribute to their development as changemakers? Given the exploratory nature of this topic and the limited existing literature, we employed a semi-structured interview format and an inductive analytic approach to allow unanticipated insights to emerge to determine the individual and organizational factors that support and enhance changemaking in attending physicians.

Methods: Qualitative semi-structured interviews were conducted with 15 physician changemakers who have successfully implemented transformative healthcare initiatives. Data were analyzed using Braun and Clark’s reflexive thematic analysis (RTA) to identify key themes and develop a comprehensive understanding of their experiences.

Results: Analysis at the individual and organizational level illuminated a constellation of interconnected themes propelling these physician changemakers: Participants’ insatiable appetite for learning, coupled with sustained inspiration and the capacity for embracing ambiguity and emotional regulation, drives changemakers’ courage and resilience. Notably, while challenging, participants did not perceive changemaking as unduly burdensome. At the organizational level, changemakers perspicaciously report leveraging the system, engage in job crafting through protected time, and authentically take accountability and own the change.

Conclusions: With an increased focus on addressing the systems issues that impact quality healthcare, this study provides a roadmap for individual and organizational actions to expand and accelerate the number of physician changemakers.

## Introduction

Less than half of the Americans characterize their healthcare as good or excellent [1]. Despite leading the developed world in per capita healthcare spending [2], the United States lags behind other industrialized countries in key healthcare quality outcomes, including life expectancy and infant and maternal mortality [3-5]. There is a growing consensus among patients, physicians, and policymakers that healthcare must undergo a massive transformation [6-8]. Recognizing this need for change, the Institute for Healthcare Improvement developed the Triple Aim (reducing costs, improving patient experience, and population health [9]), later evolving it to the Quadruple Aim by incorporating healthcare professional well-being [9]. This move acknowledged the growing literature that healthcare professional well-being is crucial to the quality of healthcare patients receive [9].

A direct threat to physician well-being is the prevalence of burnout, with approximately 50% of physicians experiencing burnout defined as exhaustion, decreased efficacy, and distancing from one’s work [10]. There is increasing recognition that system dysfunction contributes to burnout, attrition, and a decreased sense of agency among physicians [11-13]. A decreased sense of agency or efficacy, along with distancing from one’s work, is counter to the conditions needed to enact change. Decreased efficacy is closely linked to the phenomenon of learned helplessness, wherein “an individual continuously faces a negative, uncontrollable situation and stops trying to change their circumstances” [14]. George et al. describe that physicians often feel like the “glue that binds together elements of a dysfunctional system” [15]. This feeling of being the glue, coupled with little formal training on how to enact wider systems change, can drive feelings of burnout, learned helplessness, and lack of agency [15]. Thus, studying physicians successfully enacting change provides a window into activating physician efficacy to promote change.

There is a paucity of research on the individual and organizational factors that promote change in the healthcare context. Studies by Gonzalo et al. focused on internal medicine faculty [16], and Velthuis et al. explored how undergraduate medical curriculum leaders enact change [17]. The Velthuis et al. study provides an in-depth analysis of the individual transformation that must occur within the challenging milieu of modern healthcare [17]. These initial findings illuminate the need for further research to comprehensively elucidate the individual and organizational factors enabling successful change experiences.

Drawing from the organizational psychology literature, Lippit, Watson, and Westley proposed a seven-phase model reliant on an external change agent [18]. Change agents are “anyone who has the skill and power to stimulate, facilitate, and coordinate the change effort” [19]. A changemaker is someone who takes intentional, creative action to solve a societal problem [20]. For this research, the authors specifically chose the term changemaker over change agent to emphasize the focus on changes for the greater good of patients and healthcare workers. Thus, to enact the Quadruple Aim, there is an urgent need to increase the number of changemakers and shorten their learning curve. 

Kotter’s eight steps for leading change provide a helpful framework for the steps of the changemaking process [19]. In addition, papers have been published on quality improvement endeavors, adding to the tactical approach to enacting change [21,22]. The remaining gap is the connective tissue between Kotter’s eight steps. For instance, the first step is creating a sense of urgency. There is a fine line between creating urgency and creating undue individual pressure that can negatively impact the team and hinder change. Thus, it is imperative to understand the inner work that changemakers must do to undergo the transformation that leads to inspiring and equipping effective changemaking in the healthcare context. Furthermore, a rich understanding of the individual and organizational factors that equip changemakers provides a set of conditions that can be employed across a broad set of challenges. While papers describing quality improvement and other change endeavors are helpful, they can be impossible to apply within another organization without a richer understanding of the individual and organizational factors underpinning and creating the conditions that support change. 

Drawing from the change literature outside healthcare, the major organizational psychology theorists view change through the individual and organizational frames [23,24]. This literature has delineated systems thinking, improving mental models, fostering dialogue, and a culture that promotes double-loop and triple-loop or deutero learning at the individual, group, and organizational level as key components driving profound change [23-25]. Furthermore, the aforementioned reflective capabilities are said to promote profound change, “touching the innermost domains of the person (cultivating personality, core value, habits) and the collective organization" [26]. Similarly, Perkins et al. eloquently articulate that the precondition for transformative change encompasses “an organization that has both the infrastructure and the culture necessary to support the process of organizational learning….The individual is seen as an active agent and acts to intervene in organizational processes. Hence, a learning organization depends on openness to new ideas and change at both the individual and organizational levels" [27].

Combining these perspectives, the literature suggests that change requires transformative learning by the individuals and transformational change by the organization [28]. Recognition of the duality of the individual and organizational lens is important to unlocking physician agency. This progression, from focusing on individual to organizational factors that determine the well-being of both individuals and effectiveness of the healthcare organization, is reflected in the move from what is referred to as wellness 1.0 to 2.0 [29]. 

This research aims to inform future endeavors to support individuals and healthcare organizations in developing more changemakers and to shorten their learning curves. Thus, in this exploratory study, we investigated how physicians enact and experience changemaking: How do attending physicians from various specialties construct their understanding and make sense of the individual and organizational factors that contribute to their development as changemakers? What inspires and propels these physicians to be changemakers? This is a foundational step as we consider curricula to enhance changemaking along the medical career continuum. The findings from this research were previously presented as a poster at the 2024 Women in Medicine Summit™ on September 13, 2024.

## Materials and methods

This was IRB-approved qualitative research operating within a constructivist paradigm [30]. Informed consent was obtained from all interviewees prior to their involvement. Participants were informed that their participation was completely voluntary. Anonymity and confidentiality of participants were maintained, and ethical guidelines from relevant research ethics committees were followed.

Context

We aimed to authentically represent physician changemakers' reflections and experiences while acknowledging the potential influence of our own perspectives in shaping the interpretation of their narratives.

Participants

Participants included 15 physicians who led change efforts within healthcare. Participants were recruited through criterion and convenience sampling [31]. We purposely sampled participants actively involved in a successful change initiative that improved aspects of healthcare's Quadruple Aim [8]. Cost reduction was considered beneficial only if it didn't negatively impact the other three aims. During the interview process, participants were asked to describe their successful change initiatives in detail, allowing the research team to confirm that each effort met the criteria for meaningful impact across one or more domains of the Quadruple Aim. This detailed description also ensured that the participants’ experiences would offer a rich account of individual motivations and organizational dynamics involved in changemaking.

Data collection

Author 1, AU, conducted 45-60-minute semi-structured interviews. The interview protocol was refined based on one pilot study. The interview comprised two distinct components: (1) an initial exploration of a specific change initiative undertaken by the participant, including their role and the organizational context, and (2) a subsequent inquiry into their personal journey and motivations for becoming a changemaker. The interview guide (Appendix 1) was utilized, along with flexibility, to allow the interviewees to discuss their experiences and perspectives. Demographic data was collected along with institutional biographies. All interviews were conducted virtually, audio-recorded, and transcribed verbatim to ensure accuracy. Interview transcripts were de-identified, and pseudonyms were assigned to ensure confidentiality.

Data analysis

We employed Braun and Clark's reflexive thematic analysis (RTA) to explore how leaders make sense of change [32]. This framework accommodates inductive, data-driven exploration of thematic content to guide the analytical process. This flexibility allowed for a dynamic and nuanced understanding of the data. Two researchers independently coded each interview. Codes emerged organically from the data through the research teams' iterative engagement with the transcripts. Others were inspired by theoretical models focusing on change at the individual and organizational level [21,33]. Our research team iteratively embarked on a rigorous process of data familiarization, code generation, theme development, coding, and theme refinement through weekly dialogue, meaning-making sessions, and Author 1 memoing to draw out a comprehensive narrative of participants’ perspectives. This reflexive approach allowed the research team to uncover novel insights and connections to broader theoretical discourses, illuminating a comprehensive and theoretically grounded understanding of the changemaking process. Sufficiency was obtained after the tenth interview [34].

Author 1 (AU) is an emergency physician who underwent a personal transformation that resulted in a renewed commitment to changemaking with an emphasis on systems-level solutions. AU hosts a podcast focused on healthcare transformation, exposing her to individual and organizational themes related to changemaking. Author 2 (AW) is an emergency medicine physician associate and researcher who has served in healthcare and academic leadership roles. She is a leadership coach and teaches and publishes on leadership, strategic hiring, and equity. Author 3 (JH) served 25 years in military medicine in academic leadership roles and teaches and publishes on leadership topics. Author 4 (JM) is an educational psychologist who values integrative theory, the role of metacognitive reflection, emotions/emotional regulation, and complexity theory in medical education. We acknowledge that our perspectives and experiences influenced the research questions we posed and the subsequent data analysis we conducted.

## Results

The study had more women (nine) than men (six) with varied leadership roles and specialties. Participants were largely in academia at associate or full professor rank. One-third were outside academia, including community medicine, nonprofit, and consulting work. Nearly half of the participants were working toward or held an advanced degree beyond MD/DO (Table 1).

**Table 1:** Specialties, professorship, advanced degrees, and change endeavors in participants

Specialty	Fellowship-trained	Roles	Advanced degrees	Academic Rank (associate, full, or n/a)	Summary of change endeavors
Emergency medicine (7)	Pediatric emergency medicine (1)	Vice Chair (1), Assistant Dean, Chief Medical Officer (2), Executive Director (1), Consultant (1), Program Director	Master of Medical Management (1), MBA (in progress)	Full (1), Associate (2), n/a (4)	Led strategic planning for a physician organization. Founded a nonprofit for equity research for improved healthcare quality standards. Advanced recruitment of minority groups through bias training. Advocated against physician shame/errors. Prevented a hostile corporate takeover. Quality improvement in prescribing behaviors. Negotiated physician call coverage improvements
Internal medicine (5)	Infectious disease (2)	Medical Director (1), Chair (2), Curriculum Director (1), Associate Chair and Chief of Clinical Affairs (1)	PhD (1), MPH (1), Master of Medical Management (in progress)	Full (4), Associate (1)	Guided clinic recovery and COVID-19 response. Created new educational certificates and programs. Established mental health services in primary care. Founded two fellowship programs
Pediatrics (1)	Pediatric oncology (1)	Medical Director (1)	Masters in Health Admin (1)	n/a	Developed an interdisciplinary clinic for children with complex needs
OB-GYN (1)	Maternal-fetal medicine (1)	Chair (1)		Full (1)	Created an in situ simulation program for obstetrical emergencies
Family medicine (1)	0	Dean (1)		Full (1)	Built a comprehensive faculty development program for distance faculty

The themes and codes are presented in Table 2.

**Table 2:** Summary of themes and codes for qualitative data

Theme	Code
Insatiable learning	Growth mindset, feedback, continuous improvement, reflection, self-regulated learning
Inspiration and sustainment	Support system, motivation, purpose, passion
Courage and resilience for sustained change	Perseverance
Navigating the complex emotional landscape of leading change	Vulnerability, reflective awareness, positive emotions, negative emotions, emotional regulation, conflict resolution
Communicating for influence: listening for dissent and intentional messaging	Storytelling, active listening, persuasion
Leveraging the system	Policy change, institutional support, resource allocation, consensus building, collaboration
Job crafting	Autonomy
Owning change	Leadership, accountability

Eight themes were identified. The illustrative quotes corresponding to the themes are outlined below.

Individual level analysis

Insatiable Learning

Across interviews, participants consistently emphasized that becoming effective changemakers required a continual commitment to learning to acquire new skills and adapt to the complex, evolving nature of healthcare systems. Insatiable learning was characterized as an internal desire to take action to learn how to be effective changemakers. They shared a level of self-awareness and drive to close the gaps to be more effective changemakers, captured in this quote: *I want to apply [a] growth mindset… I don't have all the leadership skills that I need…but through learning on the fly, realizing where my deficit knowledge gaps were, [I] quickly threw myself into leadership training (P3)*.

After reflection, they formulated a plan to increase learning. Many were working on advanced degrees, which they attributed as key to their changemaking trajectory: *I enrolled in a master's course in medical management…because I felt stagnant after a few years of being program director…and [I] needed to enhance my professional career and growth and help people (P4)*.

All emphasized the importance of self-regulated learning through informal methods such as reading books or journal articles, listening to podcasts, or seeking formal offerings such as lectures, courses, or certificates. Others highlighted the importance of learning from peers and within networks, mentors, along with actively seeking instruction, guidance, and feedback: *More than the leadership course [content], I realized that there was a lot that could be learned from other people (P14).*

Finally, some participants articulated that they approached the change process as a learning experience and that piloting, seeking feedback, and reflecting on successes and failures provided valuable learning that expanded their thinking and shortened the learning curve to implement change.

Inspiration and Sustainment in Changemaking

Participants had varied changemaking inspirations. Many participants traced their passion for healthcare advocacy to their upbringing, citing parental influence and early life experiences as key motivators. P12 shared how witnessing negative healthcare experiences of her Black family fueled her commitment to improving equity and quality, particularly for patients of color: *The downstream effects and poor health outcomes if patients that look like me [and] my own family members, those are things that motivate me to do the work that I'm doing today.*

Similarly, P9 described: *He's sort of the measured…quiet justice, but my mom is definitely where I got fire in the belly…I think being an immigrant kid being open to a lot of different ideas, I was used to having to stand up for myself*.

For others, the impetus stemmed directly from acute crises, while others described the inherent intellectual stimulation and gratification associated with changemaking:* We were in a crisis. The clinic was in crisis. And something had to be done and something had to be different (P3).* *I think that seeing a problem and wanting to fix it is fun (P15)*.

P12, P9, and other participants described an intense drive to find solutions to improve the quality of care their patients received and the working conditions of their colleagues. While none specifically used the term moral injury, they described feelings of distress, followed by a choice to engage in change efforts. Many described outside their working hours, driven by feelings that this was crucial work, buoyed by the knowledge that they perceived as making a difference in the lives of their patients and colleagues.

Navigating the Complex Emotional Landscape of Leading Change

Participants described an emotional journey that is complex and multifaceted. They expressed experiencing excitement and optimism tempered by caution and an awareness of the potential challenges. P15 expressed fluctuating emotions: *I can feel very enthusiastic, excited, and motivated and want to see results. I could find myself very frustrated and annoyed as doors get closed…I've written many angry emails that I let sit in my email draft basket just to make myself feel better.*

However, passion, engagement, and deep dissatisfaction with the status quo also fuel their drive to make a difference: *I remember I was very angry with the people who had come before me and neglected to teach me about this very important thing…I needed to look outside of medicine to learn how to deal with it….someone told me once that my brand was righteous anger (P9).*

Most participants indicated the ability to recognize and regulate their emotions: *I'm a very human leader and I have my own emotions (P3).* *[You have] to suspend emotion…You have to love the people, and you have to love the process, and I don't mean you have to like them, either the people or the process, but you have to just be willing to get up every day and passionately engage in the work (P10)*.

Some implemented proactive strategies to manage their well-being during the intense change process: *I just kind of give myself days off…I just put a place marker in my calendar…and I don't let anybody put anything on that day, and I just go for a walk and go out to lunch… I have some mental health days (P6).*

Courage and Resilience Sustained Change

Reflectively zooming in and out, participants noted that their inspiration for change was confronted with the difficulty of leading change. They uniformly described the messy and stressful nature of changemaking. Participants consistently embraced the uncertainty inherent in change. They acknowledged the personal and professional costs, the loneliness of forging new paths, and the risk of failure. As P10 poignantly stated: *It's not easy to do, it can be very lonely trying to build something that you don't know if it's ever going to work.*

Yet, they persevered, driven by an unwavering belief in their vision and a refusal to accept "no." Similar to P8, their narratives revealed an adaptive approach to navigating setbacks, flexibly adjusting course: *It's like being able to assess each individual situation and figure out what your end goal is. I don't know that I was confident…but I'm not afraid to change.*

Participants conveyed that their ability to embrace the unknown, coupled with a strong sense of purpose and supportive relationships, fueled their success as changemakers. P5 expressed, *I was fortunate to have a couple of really stabilizing relationships…I think you can have a lot of adversity but you have to have that person that gives you perspective and hope*.

All participants were purpose-driven and articulated how either looking back on formative experiences or looking out at how the change impacted their patients, colleagues, community, and society contributed to their grit.

Communicating for Influence: Listening for Dissent and Intentional Messaging

All participants discussed the importance of bidirectional dialogue. They emphasized listening to understand and foster trust as precursors to changemaking:* I just listened to the people around me…with the end goal of just making everyone in the organization feel like they had a voice in the mission and access to leadership (P5).*

Participants voiced that it was critical to have diverse people in their sphere to hear different perspectives and to create an environment where individuals feel comfortable expressing disparate views: *You have to be intentional in bringing people with different viewpoints and to really poke out your blind spots (P1).*

Listening to diverse perspectives served as a vehicle for problem-solving by generating new ideas: *Conversations with this person pushed us to a level of thinking through all the possible downsides… if we would have not had the resistance…we would have failed because of the very thing that she called out (P10).*

Participants voiced that listening takes time and could delay decision-making but leads to more effective solutions. Participants commented on being transparent, strategic, compelling, and even entertaining when communicating, with P1 sharing that joining Toastmasters helped refine her messaging. Additionally, some emphasized multichannel communication tailored to receiver preferences and designed to cut through fast-paced, information-saturated environments.

Organizational level analysis

Leveraging the System

Leveraging the system refers to the participants’ narratives of understanding how the system works and engaging stakeholders to foster change. Most participants described needing to learn more about their system, including increasing their knowledge of organizational and accrediting bodies’ rules, processes, priorities, and regulatory laws, to effectively drive change: *One of the things in your back pocket is that every organization is ultimately accountable to some other regulatory body or employer, or funding mechanism and so sometimes identifying who the stakeholders are that have some authority to be able to help drive change and support your mission (P1)​​​​.*

Nearly all identified fiscal constraints, competing priorities, and time as barriers to enacting and sustaining change. With this in mind, they created compelling narratives to align their changemaking with the requirements and priorities of their organizations. They exhibited pragmatism, acknowledging systems limitations and the time needed to build consensus. Participants emphasized the importance of maintaining and investing in relationships to create and sustain long-term change: *Slowly building a relationship from the ground up…there are a couple people that are…not my favorite person in the world or in the workplace. But I'm still going to preserve this relationship as much as I can (P3).*

Participants stressed the value of teams for collaborative learning to energize the process: *Any good change management, you need a good team, and you need other people (P15).* *I think… finding common ground and getting the right players on board and working with passion…[and] playing to everyone's strengths, playing to my own strengths (P12).*

In addition, many participants recognized political forces within their organization and learned to proceed strategically rather than with unbridled altruism.

Job Crafting to Enhance Changemaking

All participants exhibited high levels of intentional job-crafting behaviors. They described prioritizing tasks that aligned with their passions, strengths, and desire for justice within the constraints of their assigned roles. Similarly, others described the tension between protecting time for changemaking and the need or desire to keep clinical medicine in their portfolio to ground their change efforts. P4 stated: *That's how I crafted the job …the dean role needed the lion's share of my time, but I personally wasn't ready in my career at that point to give up all clinical.*

Participants relayed the significant amount of time changemaking endeavors require: *People really don't realize how much time and work moving change forward takes (P15).*

Most participants worked less than 50% clinical, with the remaining time allocated to educational and administrative tasks that facilitated changemaking. Several participants mentioned the importance of compensation, including stipends or buy-downs, to ensure protected time to support the work. 

Along with stipends and buy-downs, several participants discussed the importance of titles and gaining leadership roles. While all participants emphasized that titles alone will not guarantee changemaking effectiveness, there was more of a trend toward women participants noting that titles were helpful with promoting credibility and increasing the likelihood of initial conversations: *I think titles are really important, and it's something particularly for women in leadership that we ask for those titles to be able to have a little bit of the same footing as some of our male counterparts in negotiating and having that mutual respect. At the end of the day, a title is just a title, and people will follow leaders not because of their title, but because of their actions and their ability to inspire and generate trust (P1)*.

In addition, one-third of participants worked primarily outside of academic institutions. It was noted that these participants were acutely aware of fiscal constraints, the importance of rapidly showing return on investments (ROIs) for the nonprofit or for-profit organizations they worked for or with. This emphasis on ROI led many to adopt an entrepreneurial approach, looking for ways to demonstrate growth, seek publicity, and form strategic relationships to advance their changemaking efforts.

Owning Change

Participants grasped the dynamic interplay of individual and organizational forces in driving sustainable change. They embraced personal accountability, exemplified by P11's statement: *We weren't always here for people. We got to own that. If it's me that's owned it, then that's okay (P11).*

This sense of ownership (of past institutional failings) extended to strategically leveraging existing structures to facilitate change while advocating for flexibility and autonomy at all levels.

## Discussion

The changemakers interviewed in this study provided insights into what inspires, equips, and sustains change within medicine, both personally and organizationally. While there has been some previous work on factors associated with changemaking in healthcare, this paper provides additional depth and details on the professional development and internal work, coupled with organizational factors that support physician changemakers [15-17,21,33]. Our study extends the medical literature by introducing a multilevel perspective of individual and institutional factors that promote the changemaking process.

This study found a universal drive among participants for continuous learning in their changemaking endeavors. Their experiences underscore the critical role of reflection in fostering self-awareness and fueling improvement. This resonates with the broader change literature and Schön's "reflection-in-action" concept, where practitioners engage in a continuous cycle of experiencing, reflecting, and learning [35]. The participants' proactive engagement in diverse learning activities exemplifies a commitment to self-regulated learning and professional growth. Velthuis et al. also support that fostering a culture of reflective practice within medicine may be crucial for cultivating changemakers [17]. A systematic review by Winkel et al. also found that reflection was important for increasing learning of complex subjects and deepening professional values [36].

Courage, resilience, and the discomfort of complicity

This study confirms that changemaking necessitates both initial activation energy and sustained effort driven by courage and resilience. While this study focused on the successful implementation of change efforts, it’s important to note that all participants describe multiple failures. Their ability to reframe and view failure as part of the process was universal among participants. Participants' narratives illustrate this evolution, moving from early enthusiasm to deep understanding of the inherent challenges. This aligns with Ebrahimi Ghassemi et al.'s framework, which emphasizes the teachable nature of courage through self-advocacy, bravery, and persistence. Participants demonstrated resilience (optimism, competence, strong relationships) as they ethically grounded their changemaking efforts, further supporting Ebrahimi Ghassemi et al.'s model for effective social change leadership [37], motivated by a desire to improve patient care and support colleagues, often requiring navigation of emotional challenges and systemic barriers. Many participants were driven by a sense that they couldn’t care for patients in the way they felt was right. This sense of injustice, and the discomfort of feeling complicit in the system, has been described in the healthcare context by Dean as moral injury [13]. These participants clearly articulated that their changemaking efforts reduced the distress that many experienced.

The ubiquity of emotions in changemaking

Our findings resonate with Ratnapalan et al.'s work, which highlights the complex interplay of emotions experienced by changemakers. Our study similarly observed a range of emotions among participants, coupled with an ability to navigate this complexity. Ratnapalan et al. suggest that the change process itself can serve as an outlet for regulating challenging emotions like frustration and anger, also supported by our participants' experiences [38]. Notably, they exhibited reflective awareness of their emotions and recognized their potential impact on others. This awareness translated into active emotional regulation, such as strategically choosing when and how to express emotions, including proactive approaches, as illustrated by the examples of unsent emails and blocking out schedules.

Participants consistently demonstrated the ability to observe, monitor, and regulate their own cognitive and emotional processes, aligning their actions with personal and organizational values. This resonates with Merkebu et al.'s definition of metacognitive reflection, which emphasizes internal observation, contextual awareness, and regulation of knowledge, experiences, and emotions [39]. Cultivating metacognitive skills through reflective practice and mindfulness training may be valuable for developing more effective changemakers (Table 3).

**Table 3:** Organizational strategies to support changemaker physicians

Theme	Suggested organizational actions to support changemaking
Insatiable learning	Funding, encouraging, and incentivizing attendance at lectures, seminars, certificate programs, advanced degrees, courses, and conferences. Mentorship programs
Inspiration and sustainment in changemaking: courage and resilience	Coaching programs. Peer support. Just culture. Promoting the development of diverse networks. Sponsorship. Appropriate staffing and resources to encourage reasonable workloads, vacations, and rest between scheduled work
Navigating the complex emotional landscape of leading change	Peer support programs. Access to mental health. Appropriate staffing and resources to encourage reasonable workloads, vacations, and rest between scheduled work. 360 evaluations to encourage professional growth in emotional domains. Professional development to enhance emotional regulation. Metacognitive reflection and mindfulness training. Narrative medicine programs. Schwartz rounds
Communicating for influence: listening for dissent and intentional messaging	Professional development on communication and messaging, including leveraging innovative tools, such as AI to generate impactful and engaging messaging. Public relations training. Feedback mechanisms for public speaking to assist with professional development. Reflective practice and bias awareness
Leveraging the system	Onboarding to ensure systems awareness regarding policies, procedures, and how change occurs AI-enabled meeting notes, summaries, and standardized processes for distributing meeting summaries and tracking projects. Transparency on processes. Clear vision and mission, along with priorities, communicated to all staff. Appropriate support staff to facilitate change efforts and use physician time effectively. Formalized and protected time to promote quality hand-offs and succession planning and development of future leaders

Furthermore, this study underscores the bidirectional relationship between individual emotional regulation and organizational culture. While acknowledging the influence of organizational culture on emotional expression, it also highlights the potential for individual transformations to catalyze cultural change. Our findings align with Cote et al.’s research on the social effects of emotion regulation in organizations [40]. Participants demonstrated healthy emotion regulation, suggesting that fostering these skills at the individual level can contribute to positive change within teams and organizations.

Moreover, we found a strong connection between emotional investment and effective physician changemakers. Participants' narratives emphasized a deep sense of caring for both the work and the individuals involved. This caring manifested through active listening, valuing diverse perspectives, and fostering trust and psychological safety. These findings align with Nembhard et al.’s research, which demonstrates the positive impact of inclusive leadership and psychological safety on quality improvement efforts [41]. The participants' ability to integrate divergent opinions and build coalitions further highlights their sophisticated emotional regulation and metacognitive skills. By creating an environment where diverse voices are heard and valued, these leaders foster a sense of shared purpose and collective ownership.

A labor of love: the passion behind changemaking in medicine

The use of terms like "love the people" and "love the process" suggests a deep passion and emotional connection to their changemaking work. This finding adds another dimension to the understanding of change leadership in medicine. This goes beyond mere professional duty, indicating a genuine affection for the individuals involved and the collaborative journey of change. It’s important to note these individuals did not perceive the changemaking process as unduly onerous. This affective commitment likely contributes to their resilience and motivation in navigating challenges and setbacks. This study suggests that cultivating emotional bonds and promoting inclusive leadership practices are crucial for developing effective changemakers in medicine.

Changemaking in imperfect systems

Finally, our study highlights the crucial interplay of individual agency and organizational support for successful changemaking. While participants acknowledged their organizations' inherent complexities and imperfections, they also believed in their ability to effect change and contribute to a shared, noble mission. Rather than being defeated by bureaucracy or complexity, they exhibited insatiable learning, coupled with learning how to leverage the system to enact change. This suggests that learning how the system works and how to move the levers of change is key to supporting the efficacy of changemakers. This ability to adapt their mindset to the organizational reality is supported by a framework suggested by Van de Ven and Sun [42]. Empowering individuals to become effective changemakers requires organizations to foster a culture that values individual contributions, provides tangible support (e.g., grants, protected time) for change initiatives, and allows for job crafting to align passions with organizational goals. Furthermore, creating a psychologically safe environment with opportunities for learning from failures, mentorship, and early career support empowers individuals in their changemaking endeavors [18].

While Kotter’s eight steps for leading change provide a helpful framework for physician changemakers, the themes that emerged in this study provide valuable insights into the personal transformation during the change process in the healthcare context [43]. It is increasingly recognized that systemic issues, including moral injury, underpin burnout and attrition [13]. The findings of this study serve as a departure point to think critically about how to support everyday physician changemakers at the individual and organizational level.

Limitations and future directions

Our study is not without limitations. Most significantly, the study is subject to recall bias. In addition, examining these perspectives with interviews with stakeholders, peers, and supervisors would provide additional information and deeper insights. This study did not explore the resources or funding for changemaking. Both community and academic participants described a significant amount of protected time, and more studies are needed to provide a fiscal strategy, especially given the extreme financial challenges facing healthcare.

This was a qualitative study of 15 attending physician changemakers, and not all specialties and career stages were represented. The study included more physicians practicing in academic than community settings. In addition, all were mid to late career; thus, it is important to exercise caution when applying findings to early career professionals. Of note, emergency medicine was overrepresented in the sample. However, given this specialty with the highest rates of burnout, understanding changemaking in this group with a higher risk for reduced efficacy may offer additional insights to enacting change in difficult settings. The study was not powered to explore differences in changemaking among various demographic groups, and thus, there may be important differences among genders, specialties, and career stages. 

Importantly, this study did not seek to explore how moral injury related to changemaking, yet this concept emerged during the data analysis. Participants described feelings of moral injury and linked their change efforts, in part, to decreasing their own distress. We did not measure burnout in the study participants, and future studies exploring burnout rates, including a mixed methods approach to provide qualitative comments linked to the quantitative burnout data or moral injury scores, are important future studies.

We have highlighted the importance of individual transformation that leads to team and organizational change. Future research should investigate how academic versus nonacademic roles shape changemaking approaches, exploring the distinct strategies and priorities of changemakers in these diverse professional contexts to inform tailored support and interventions. Some organizations may require a significant overhaul to be a psychologically safe place for changemakers to flourish. Changemaking can be risky, as creating change involves acknowledging that the status quo is insufficient [44]. There are no clear changemaking curricula to support the development of changemakers throughout a career in medicine. Changemaking journeys are inherently unique; the themes identified, paired with actionable examples in Table 3, offer a roadmap that individuals and institutions can adapt to their own context to support sustainable transformation. While research is needed, this study highlights the potential actions that organizations can implement to foster an environment that supports and accelerates physician changemakers.

## Conclusions

Changemaking in medicine is complex. These physician changemakers demonstrated a remarkable combination of emotional regulation strategies coupled with unwavering commitment and genuine passion for the people involved and the process itself. The study illustrates the power of supportive cultures to foster or hinder change. Lessons from this paper provide tangible ways to inspire and equip physician changemakers to accelerate positive change in medicine.

## Appendices

Interview guide

The purpose of this study is to explore the individual and organizational factors that support changemakers in healthcare. You have been identified as a healthcare changemaker, which is someone who takes intentional, creative action to solve a problem. At any point during this interview, we can stop, and you are free to withdraw.

Questions

Tell me about your current role and path to that role.

Follow-up questions: Describe your clinical specialty, administrative or educational roles, and percentage of time clinical vs nonclinical?

Can you describe a change you’ve made in healthcare in as much detail as possible?

Additional questions:

Tell me more about the change.

Describe the setting that this change occurred in, for instance, a clinic, hospital, state, or organization, and how many people you think were impacted.

Can you describe your role (e.g., more active/ passive, proactive/reactive) during this process?

What inspired you to initiate this particular change?

How did it happen?

As the change was unfolding, what were your general perceptions, emotions, and thoughts accompanying the circumstances of this event?

Did you feel confident you could be successful in enacting the change?

Why?

In thinking about your official title or position,  what are your perceptions about the degree to which your positional authority was (or was not) helpful in enacting this change?

What are your perceptions about the conditions that supported you to make this change?

What particular obstacles did you encounter, and what kept you driving forward despite the adversity?

What did your leaders do to support your change effort?

Thinking back on the organization/setting that this change occurred in, were there any organizational or system-wide practices that supported the enactment and sustainment of the change?

Can you describe the overall culture (including rules, norms, decision-making process, top-down v. bottom-up, communication process, etc.) of your organization and how that might have bolstered or obstructed the change process?

Question break

The next set of questions will focus more on your journey to becoming a changemaker.

Reflecting on your life experiences and career, how did you become a changemaker?

Who helped enact and sustain the change? What specifically did they do to help? Was there anything that anyone did that hindered change efforts?

Do you always seek to make changes, or was there something specific to this situation that spurred you to make the change?

Follow-up prompt (if not addressed in the previous question):

To what extent do you consider yourself an active change agent?

If not addressed, does it extend beyond medicine?

What or who inspired you to be a changemaker?

Reflecting on your life experiences and career, how did you become a changemaker? The how refers to any courses, books, or experiences that gave you the knowledge, skills, and attitudes to be a changemaker.

Is there anything in your past that, looking back, particularly prepared you to overcome setbacks, challenges, and adversity inherent in the change-making process?

We talked about many things today. Is there anything else related to changemakers that you’d like to add?

After today’s session, would it be okay if I send you a brief follow-up email so you’ll have my contact information in case you think of something you’d like to add to our discussion today or have any questions. Also, if after today’s session, you’d like to withdraw from the study, you can do that by letting me know or contacting anyone else listed in the follow-up email. Thank you for participating!
